# Using Abbreviated Injury Scale (AIS) codes to classify Computed Tomography (CT) features in the Marshall System

**DOI:** 10.1186/1471-2288-10-72

**Published:** 2010-08-06

**Authors:** Mehdi M Lesko, Maralyn Woodford, Laura White, Sarah J O'Brien, Charmaine Childs, Fiona E Lecky

**Affiliations:** 1 University of Manchester, Manchester Academic Health Science Centre, the Trauma Audit and Research Network (TARN), Salford Royal NHS Foundation Trust, Salford, UK; 2 University of Manchester, Manchester Academic Health Science Centre, Occupational and Environmental Health Research Group, Salford Royal NHS Foundation Trust, Salford, UK; 3 National University of Singapore, Yong Loo Lin School of Medicine, Singapore; 4 University of Manchester, Brain Injury Research Group, Salford Royal NHS Foundation Trust, Salford, UK

## Abstract

**Background:**

The purpose of Abbreviated Injury Scale (AIS) is to code various types of Traumatic Brain Injuries (TBI) based on their anatomical location and severity. The Marshall CT Classification is used to identify those subgroups of brain injured patients at higher risk of deterioration or mortality. The purpose of this study is to determine whether and how AIS coding can be translated to the Marshall Classification

**Methods:**

Initially, a Marshall Class was allocated to each AIS code through cross-tabulation. This was agreed upon through several discussion meetings with experts from both fields (clinicians and AIS coders). Furthermore, in order to make this translation possible, some necessary assumptions with regards to coding and classification of mass lesions and brain swelling were essential which were all approved and made explicit.

**Results:**

The proposed method involves two stages: firstly to determine all possible Marshall Classes which a given patient can attract based on allocated AIS codes; via cross-tabulation and secondly to assign one Marshall Class to each patient through an algorithm.

**Conclusion:**

This method can be easily programmed in computer softwares and it would enable future important TBI research programs using trauma registry data.

## Background

Trauma registries hold records of patients with Traumatic Brain Injury (TBI) across a designated region mainly for assessment of trauma care centres/systems compared with a national standard e.g. analysing data to predict survival probability (observed - expected survival rates). The demographic and clinical details of trauma patients are submitted to these registries primarily to provide data that will improve clinical outcome for trauma patients but they also form a valuable dataset for epidemiological studies. The Abbreviated Injury Scale (AIS) [1,2] was proposed by the Association for the Advancement of Automotive Medicine and was designed specifically for coding various types of injury and for scoring them based on the severity. Using a standard dictionary, each entry in a trauma registry dataset is assigned a 6-digit AIS code number with a post decimal place representing score of severity. The description for each AIS code is contained in the AIS dictionary. Each post-decimal score of the injury severity ranges from 1 (minimal) to 6 (maximal).

The AIS dictionary is structured by anatomical region of the body such as face, neck, abdomen and pelvic contents etc. One section in this dictionary is allocated to head trauma, which is subdivided into the whole area (massive destruction of cranium and brain, penetrating injury and scalp injury), intracranial vessels, cranial nerves (cranial nerves I to XII), internal organs and skeletal. This part of the AIS dictionary contains information about the anatomical location of the lesion (brain stem, cerebrum and cerebellum), the type of the lesion (e.g. haemorrhage, contusion and brain swelling), various subtypes of haemorrhage such as Subarachnoid Haemorrhage (SAH), Subdural Haemorrhage (SDH) and the size of the lesion.

Using the AIS dictionary to describe injuries is probably limited to those running trauma registries. It is rarely employed for clinical and therapeutic purposes or in data collection for clinical trials because a trained coder is needed to code the injuries and also because the description and classifications of injuries is more detailed than required for clinical purposes. Alternatively, the Marshall Classification of structural brain damage is based on CT findings of TBI patients [3]. This system was first introduced in 1991 and the main aim was to identify those TBI patients at higher risk of deterioration or mortality. This classification has been validated as having predictive value for TBI outcome as well [4-7]. The hierarchy of Marshall Classes represents the increasing risk of developing raised ICP determined by factors relating to this pathology such as mass lesions or brain swelling. This classification challenged the previous perception that patients with compressed or absent cisterns who had a good clinical evaluation could be treated as if their brain CT is normal [3].

Understanding the relationship between AIS coding of brain injury and the Marshall Classification is important for several reasons. First, the AIS and Marshall Classification systems describe slightly different things. The Marshall Classification provides the opportunity to identify a subset of TBI patients at risk of developing intracranial hypertension. It ignores brain stem and cerebellar injuries, which are described in detail in the AIS dictionary. Secondly, the Marshall System is focused on closed head injury and was not designed for penetrating head injuries, for which there are several AIS codes. Since TBI in trauma registries tends to be coded using the AIS dictionary and in clinical settings using the Marshall Classification, it is impossible to generate a complete picture of TBI incidence, risk factors and outcome without being able to bring these two types of data together.

Therefore we propose a method for allocating a Marshall Class to the AIS codes that are recorded for a given TBI patient. We have assumed that each injury description in the AIS dictionary can be used as an alternative to the CT reports.

## Methods

### AIS coding

Coding of brain injuries in the AIS dictionary is based on anatomical location (the brainstem, the cerebellum, the cerebrum and the pituitary), the type of injury *(penetrating injury, diffuse axonal injury, contusion, hemorrhage, brain swelling, infarction, ischemia, pneumocephalus, laceration, compression, massive destruction(crush), transection)*, subtypes of hemorrhage *(epidural, intraparenchymal, subdural, subarachnoid, subpial) *and the degree/extent of the injury. Some types of injuries relate to certain locations of the brain; these being *massive destruction (crush) *which can affect the whole head or can occur in the brain stem, *compression and transection *exclusively occurring in the brain stem and *pneumocephalus *exclusively occurring in the cerebrum. However there are some other types of injuries incurred in more than one anatomical location namely *ischemia, brain swelling or various subtypes of hemorrhage *which may occur in the cerebellum or the cerebrum. Similarly, *penetrating injuries, diffuse axonal injury, contusion, hemorrhage, infarction or laceration *can be potentially sustained in all parts of the brain which include the brain stem, the cerebellum or the cerebrum. The determinants of the degree/extent of each injury include *multiplicity, being uni/bilateral *and *midline shift *(for contusions) and *the volume/diameter *(for contusions and various subtypes of hemorrhage). The severity of brain swelling in the cerebrum is determined by *the status of ventricles or the brain stem cisterns *- either or both may be compressed or absent. Where information is not adequately documented, the codes referred to as 'Not Further Specified; NFS' are assigned. Alongside the injuries which fall under the heading of 'internal organ' in the head section of the dictionary, there are codes which relate to the skeleton and some of them include descriptions of *basal skull fracture *or *not simple vault fractures*, which should, in fact, be considered as traumatic brain injury. Nevertheless, the AIS code 116002, allocated to superficial penetrating injury to the head, should be interpreted as not accompanied by brain injury. It should be noted that TBI cases may be allocated more than one AIS code.

### The Marshall Classification

Table 1 displays the Marshall CT Classification. According to this system, the discriminative features are presence/absence of intracranial pathology, presence/absence of high or mixed density mass lesions, signs of raised intracranial pressure which is status of basal cisterns and midline shift and lastly evacuation of mass lesions. In this classification, a high or mixed density mass lesion implies contusion or hemorrhage. The extent of the lesion is determined by its volume, the cut-off being 25 cc. Moreover, depending on the size and surgical evacuation, a lesion can be one of Mashall Classes II, V or VI. The higher risk of raised ICP is determined by present, absent or compressed basal cisterns and the degree of midline shift - the cut-off point being 5 mm. These pathologies fall into classes III or IV based on the severity. Unlike AIS coding, the Marshall System is mutually exclusive in that a TBI case is only allocated to one Marshall Class.

**Table 1:** The Marshall CT Classification

Marshall Class		Description
Class I	Diffuse injury I (no visible pathology)	No visible pathology seen on CT scan

Class II	Diffuse injury II	Cisterns are present with midline shift 0-5 mm and/or: lesion densities present no high- or mixed-density lesion > 25 cc may include bone fragments and foreign bodies

Class III	Diffuse injury III (swelling)	Cisterns compressed or absent with midline shift 0-5 mm, no high- or mixed-density lesion > 25 cc

Class IV	Diffuse injury IV (shift)	Midline shift > 5 mm, no high- or mixed-density lesion > 25 cc

Class V	Evacuated mass lesion	Any lesion surgical evacuated

Class VI	Non-evacuated mass lesion	High- or mixed-density lesion > 25 cc, not surgical evacuated


### Cross-tabulation of AIS codes with Marshall Classes

As explained above, the Marshall System and the AIS coding hold two different approaches to brain injury classification and thus reconciliation between the two systems required various assumptions which had to be agreed upon from both the clinical and the coding perspective. A number of meetings were held with participation of two physicians specialising in emergency medicine and neurosurgery and two experts in AIS coding in the UK (from the Trauma Audit and Research Network (TARN) [8]) to discuss the most appropriate Marshall Class allocated to each AIS code performed through cross-tabulation. Table 2 presents the resulting cross-tabulation based on expert consensus where the description for each code can be found in the AIS document. The mapping was decided to be performed initially on AIS dictionary; update 98 which is still in widespread use despite the new update introduced in 2005. Subsequently, adaptation of this cross-tabulation to suit the AIS dictionary; update 2005 was discussed (Table 3). Likewise, the decision was made to consider only AIS codes which are either apparently brain injuries (such as SAH) or, with a high likelihood, can be regarded to be accompanied with brain injury (such as basal skull fractures). However, codes relating to unconsciousness were excluded from this cross-tabulation since these codes are commonly not used by trauma registries and instead, Glasgow Coma Scale (GCS) with the same value for outcome prediction is used to address the level of consciousness [9,10].

**Table 2:** Proposed Marshall Class - AIS code combinations based on the 1998 update of the AIS dictionary

AIS codes	Marshall Class
113000.6	V/VI

116004.5	Penetrating injury

140299.5	Brain stem injury

140202.5	III

140204.5	Brain stem injury

140206.5	Brain stem injury

140208.5	Brain stem injury

140210.5	Brain stem injury

140212.6	Brain stem injury

140214.6	Brain stem injury

140216.6	Penetrating injury

140218.6	Brain stem injury

140499.3	Cerebellar injury

140402.3	Cerebellar injury

140403.3	Cerebellar injury

140404.4	Cerebellar injury

140405.5	Cerebellar injury

140406.5	Cerebellar injury

140410.4	Cerebellar injury

140414.4	Cerebellar injury

140418.4	Cerebellar injury

140422.5	Cerebellar injury

140426.4	Cerebellar injury

140430.4	Cerebellar injury

140434.5	Cerebellar injury

140438.4	Cerebellar injury

140442.4	Cerebellar injury

140446.5	Cerebellar injury

140450.3	Cerebellar injury

140458.3	Cerebellar injury

140462.3	Cerebellar injury

140466.3	Cerebellar injury

140470.3	Cerebellar injury

140474.4	Cerebellar injury

140478.5	Penetrating injury

140699.3	II

140602.3	II

140604.3	II

140606.3	II

140608.4	V/VI

140610.5	V/VI

140612.3	II

140614.3	II

140616.4	V/VI

140618.5	V/VI

140611.3	II

140620.3	II

140622.3	II

140624.4	V/VI

140626.5	V/VI

140628.5	II

140629.4	II

140630.4	II

140632.4	II

140634.5	II

140636.5	V/VI

140638.4	II

140640.4	II

140642.4	II

140644.4	II

140646.5	II

140648.5	V/VI

140650.4	II

140652.4	II

140654.5	II

140656.5	V/VI

140660.3	III

140662.3	III

140664.4	III

140666.5	IV

140676.3	II

140678.4	II

140680.3	II

140682.3	II

140684.3	II

140686.3	II

140688.4	II

140690.5	Penetrating injury

140799.3	II

150200.3	I

150202.3	I

150204.3	I

150206.4	I

150404.3	I

150406.4	I

150408.4	I

**Table 3:** Allocating a Marshall Class to AIS code; update 2005

Code	Marshall Class
140605	II

140613	II

140621	II

140625	II

140627	II

140631	II

140639	II

140643	II

140645	II

140647	II

140649	II

140641	V/VI

140651	II

140655	V/VI

140687	II

140686	II

140691	Penetrating injury

140692	Penetrating injury

140689	II

140701	I

140702	I
140703	I

140675	II

140677	II

140681	II

140683	II

140694	II

140695	II

140697	II

140698	II

150000	I

The rational for mapping various AIS codes of brain injury to the appropriate Marshall Class particularly regarding the assumptions made for brain swelling and mass lesions is provided in the Appendix.

### Selection of one Marshall Class

A TBI patient may receive more than one AIS code whereas each patient should receive only one Marshall Class in the Marshall System. In order to address this, the decision was made to place all AIS codes which fall under the same Marshall Class together as *'Equivalent to one Marshall Class'*. In this manner, Equivalent to Marshall Class I, II, III, IV or V/VI each respectively represents Marshall Classes I, II, III, IV and V/VI. Then an algorithm was devised to choose one Equivalent to Marshall Class which would be the final single Marshall Class mapped. Using of such algorithms for patients who sustained multiple brain injuries was proposed by Maas et. al. [6].

## Results

### The proposed method to allocate a Marshall Class to a TBI patient

This involves two stages: assignment of Equivalent to Marshall Classes and then selection of the final Marshall Class.

#### Stage 1: Assignment of Equivalent to Marshall Classes

Table 4 presents various AIS codes which all come under one similar Marshall Class (Equivalent to Marshall Class I, II, and III etc.). According to this table, the unclassified codes relating to brain stem, cerebellar and penetrating injuries were broken down further into penetrating, brain stem/cerebellar codes necessitating addition of two further classes of VII and VIII to represent penetrating and the brain stem/cerebellar injuries respectively. This has been agreed by the authors of previous guides for using the Marshall Classification [5] (personal communication). The other possible options are to further split the brain stem/cerebellar injuries into two distinct individual Marshall Classes or or to merge all penetrating, brain stem and cerebellar codes into one class as 'unclassified'. This depends on the research objective.

**Table 4:** Grouping of AIS codes into various 'Equivalent of Marshall Classes'

	AIS codes
Equivalent to Marshall Class I(no visible pathology)	150200,150202,150204,150206, 150404,150406, 150408

Equivalent to Marshall Class II	140602,140604,140606,140612,140614,140611,140620,140622, 140628,140629,140630,140632,140634,140638,140640,140642, 140644,140646,140650,140652,140654,140684,140688, 140686, 140699, 140676, 140678, 140680, 140682, 140799

Equivalent to Marshall Class III(swelling)	140202, 140660, 140662, 140664

Equivalent to Marshall Class IV(shift)	140666

Equivalent to Marshall Class V/VI	140608,140610,140616,140618,140624,140626,140636,140648, 140656, 113000

Cerebellar/brain stem injuries	140204,140206,140208,140210,140212,140214,140218,140299, 140402,140403,140404,140405,140406,140410,140414,140418, 140422,140426,140430,140434,140438,140442,140446,140450, 140458,140462,140466,140470,140474,140499,

Penetrating injury	140216, 140478, 140690, 116004

#### Stage 2: Selection of the final Marshall Class

**Figure 1:**
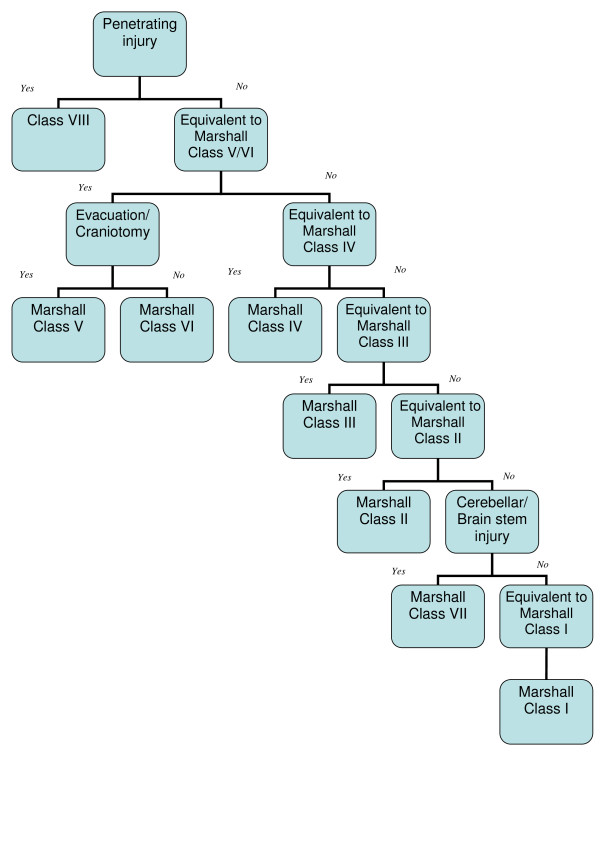
**Algorithm to derive the Marshall Class from Equivalent to Marshall Classes**.

Figure 1 displays an algorithm proposed to select one Equivalent to Marshall Class which can be the mapped final Marshall Class for a given patient. This is based on the fact that the Marshall Classification is ordinal indicating that, in case of multiple injuries, the highest class is the single class allocated to the patient. This is reflected in the algorithm. Initially, all penetrating injuries are contained in Class VIII. This is the point at which the algorithm stops since the Marshall Classification is designed for blunt injuries. At the second step, injuries are screened for Equivalent to Marshall Class V which will result in a class VI designation in case of surgical evacuation or, otherwise, class V. The following steps sequentially take account of Equivalent to Marshall Classes IV, III and II. However, prior to searching for Equivalent to Marshall Class II leading to allocation of class II, Marshall Class VII is mapped in case of the presence of brain stem/cerebellar codes. The algorithm is flexible with the position of this step being implemented prior to screening for Equivalent to Marshall Class I as displayed in figure 1 or otherwise being placed following exclusion of penetrating codes. In the latter situation, the algorithm begins its detection of the single mapped Marshall Class by exclusion of those who have sustained penetrating, brain stem or cerebellar injuries.

Programming the procedure to designate a single Marshall Class to a given TBI case for which various AIS codes are recorded is straightforward in computer softwares such as Statistical Package for the Social Sciences (SPSS) etc. In the first step, all Equivalent to Marshall Classes are computed as nominal variables for each TBI case. Each Equivalent to Marshall Class will then be 'yes' if at least one of the AIS codes allocated to this code (Table 4) is present and otherwise such Equivalent to Marshall Class is 'no'. Second, the computer has to search all computed Equivalent to Marshall Classes step by step in accordance with the algorithm. For example, if a given case has brain stem/cerebellar injuries and Equivalent to Marshall Class V/VI with surgical evacuation, the Marshall Class VI is allocated.

## Discussion

In this study, we have attempted to propose a method to translate the head injury AIS codes into the Marshall CT Classification. This involves two steps; first to cross-tabulate various AIS codes with the Marshall Classes and secondly to select the single Marshall Class allocated to a case of TBI through an algorithm. In order to perform this transformation some assumptions had to be made.

### Limitations/assumptions

Although both the Marshall Classification and the AIS dictionary group CT features according to their severity, one important difference between the two systems relates to their purposes. The main aim of the Marshall Classification is to identify those TBI patients who are at higher risk of deterioration or mortality, whereas the AIS scoring system is used to classify injuries based on their anatomy rather than physiological merits. These different approaches to CT classification mean that certain assumptions have to be made when trying to reconcile the two systems. Ideally the two systems would be completely interchangeable and no assumptions would be required. Since this is not the case an important question is whether or not mapping AIS codes onto the Marshall Classification is worthwhile. We believe adoption of the conceptual approach we have proposed allays some concerns in that, instead of strictly meeting the definition of each Marshall Class, the objective and rational surrounding that class are also employed to spot the appropriate AIS codes. A disadvantage of the Marshall Classification is that it is not a reliable classification to be used in the retrospective research settings in which the access to the real CT obtained during acute phase of therapy is often not possible in case the Marshall Class is not recorded in the existing dataset.

The Marshall Classification should be ideally performed by the expert who views the CT. However, Marshall Class II, unlike other classes, contains a broad range of heterogeneous injury types or severities. Considering the different objective of AIS dictionary which is to anatomically classify injury severities, mapping AIS codes with Marshall Classes is in fact alike ignoring many valuable individual pieces of information by pooling them into one class such as class II. This leads to them all being treated similarly in prognostic analysis despite potentially having varied individual prognostic merit on their own. This defect is substantial when class II contains, for instance, infarction beside laceration which are different in nature and perhaps in prognostic strength.

The method proposed in this study is based primarily on the assumption that the descriptions in the AIS dictionary can be substitutes for CT reports, but this is not always the case. As well as including CT reports, the sources of information to document injury descriptions also encompass MRI, surgery, x-ray, angiography, post-mortem examinations or clinical diagnosis. Nevertheless, it seems reasonable to assume majority, if not all the information for AIS coding, is obtained from CT scans since CT is the commonest modality for diagnosing structural brain damage for every patient suspected to have sustained severe trauma in the developed world and several other developing nations.

As AIS coding does not rely only on one CT report, the dynamic nature of brain injury as to progressing or regressing over time (which results in the evolution of CT findings) is reflected in AIS codes unlike the Marshal Classification. This is because the Marshal Classification is collected from CT images/reports at a certain point in time (oftentimes on admission) whilst AIS codes contain information after discharge or death. As such, using our algorithm to obtain the Marshal Class with AIS codes intermediation would inherit the information on dynamic nature or CT findings evolution as well. This poses a problem since evolution of structural brain damages per se is a prognostic factor indicating higher chance of unfavorable outcome [11]. Servadei et. al. have shown that the worst CT classification has more prognostic value than less severe CT classification (s) [11]. Consequently, if the Marshal Classification is obtained from AIS codes, there may be some overestimation of its negative prognostic role in TBI as compared to the Marshal Classification using CT images/reports. This may be particularly an issue with patients who sustain more severe brain injuries as they are more subject to various means of investigations such as MRI or operation.

As well as the above fundamental assumption regarding AIS descriptions as substitutes for CT reports, there are two other important assumptions related to the brain swelling and the mass lesion. Unfortunately neither the Marshall Classification nor the AIS dictionary describe precisely the severity of brain swelling. The degree of swelling in AIS dictionary is only determined by cistern/basal cisterns status whereas the degree of midline shift is also an important determining factor. Likewise, although midline shift or cisterns status is important in the Marshall Classification of brain swelling, other causes of midline shift, such as mass lesion, are disregarded. With respect to the size of mass lesions, future research is required to determine the precise size cut-offs for categorising such lesions, in spite of the already-known fact that larger lesions are associated with poorer outcome [12]. Comparing the cut-offs, those for subdural and epidural haemorrhage in the AIS dictionary are larger than those in the Marshall Classification by 25 cc although this difference may be negligible for contusion and intracerebral haemorrhage which is only 5 cc. The evidence base for lesion size in both classifications appears to be limited, despite claims that the cut-offs are backed by substantial experience and are not merely arbitrary [5,13].

In Table 3, we assumed that codes indicating hypoxic or ischemic brain damage are related to normal CT scan. This may not always be the case as some patients may develop brain swelling as a secondary damage to hypoxia/hypotension. Whilst our assumption of normal CT for hypotension/hypoxia may not be acceptable in our cross-tabulation, we believe the algorithm would address this problem. For example, if a patient develops brain swelling following hypoxia, then two codes of hypoxia and brain swelling will be allocated. As the injuries are taken through the algorithm, the brain swelling Marshal Class of III or IV (depending on the severity) is allocated prior to the final step of the algorithm as Marshal Class I (normal CT).

The position of various steps of the algorithm is based on the assumption that the Marshal Classification is ordinal in severity. Despite being the case from Class I to IV, this is not true for class IV versus class V as patients in class IV demonstrate lower likelihood of favorable outcome or survival than those in class V [5,14]. Since the Marshall Classification is mutually exclusive, it is conspicuously necessary to prioritise which type of injuries is more relevant for allocation of the proper Marshal Class in case of multiple brain injuries. Whilst, according to the adverse outcome frequency, the brain swelling with compressed cisterns may have to be placed prior to mass lesion in the algorithm, we believe the current position of each step is more reflective of what occurs in real life of Marshal Classification through observing the actual CT. In fact, the current position of various steps of the algorithm are according to what has been suggested by Mass et. al. [5]. In Mass's algorithm, the Marshal Classification was taken as ordinal but still class V represented lesser degrees of brain injury than class IV in their subsequent prognostic analysis.

### Implication

The Marshall Classification has prognostic value to make predictions on the outcome of the TBI patient [4-7]. AIS coding is also important from prognostic viewpoint [15] but the severity scores (ranging from 3 to 6 in TBI) encompass a wide variety of different injuries that the relationship of the score and CT findings can not be easily made particularly in clinical settings where the AIS dictionary is not a familiar tool. Hence, it is important for trauma registries to ` avoid exclusive reliance on AIS coding for the sake of better communication with the clinical audience. As the Marshall Classification holds comparable prognostic value to age, GCS, pupillary reactivity, SAH etc. [4,7] and trauma registries commonly do not have record of this classification, the translation of AIS codes to the Marshall System opens up the possibility for multivariate prognostic analysis of large series of TBI subjects saved in trauma registries. In fact, the internationally known IMPACT prognostic models [7] in TBI employ the Marshall Classification for outcome prediction and using our proposed translation not only permits running the IMPACT models in trauma registries, derivation of new prognostic models including the Marshall Classification becomes feasible. Furthermore, as other TBI series accrued in clinical studies (observational or clinical trials) often do not have AIS coding, our proposed translation facilitates mergence of datasets from trauma registries and clinical studies to conduct more powerful studies or performance of comparative analysis across datasets when data recording is not uniform.

### Future direction

The design of the algorithm is such that at the end of the allocation, there must be no cases left with no Marshall Class assigned. We tested this in a dataset of 802 TBI cases from the Trauma Audit Research and Network (TARN) with positive results (unpublished data). However, we acknowledge that our proposed allocation still requires three possible forms of validation in the future. First, it is yet to be determined how accurate our method is when the Marshall Classification is performed using the AIS codes. In this manner, AIS codes are applied as substitutes for CT reports and in case all the assumptions are followed, 100% accuracy should be met. The second form of validation is when the allocation is performed with actual CT images at hand. In this manner, the allocations are compared across two groups. In one group, the classification is done through observing the CT and in the other group the Marshall Class is obtained following assignments of AIS coding and subsequently using our proposed cross-tabulation and algorithm. This form of validation is not expected to yield 100% accuracy and it examines how strong the assumptions are. The third form of validation is to compare the Marshall Classification at certain time point with that collected from AIS codes obtained from any available source including CT, MRI, operation notes etc. This form of validation would examine the influence of multiple sources of information or the temporal effect of events on the cross-tabulation and algorithm.

## Conclusion

Using robust assumptions, we have proposed a method to allocate a single Marshall Class to a patient whose AIS codes are available, such as in trauma registries. This would enable future important TBI research programs.

## Competing interests

The authors declare that they have no competing interests.

## Authors' contributions

M.M.L. and F.L. jointly developed the idea and designed the stages of the proposed method. M.M.L. drafted the cross-tabulation and the manuscript. M.W. and L.W. provided input for the cross-tabulation from the AIS coding perspective and approved the final revision of the cross-tabulation. S.O. and C.C. reviewed the proposed method and edited the final draft of the paper to ensure content accuracy. F.L. provided input from the clinical perspective, approved the final revision of the cross-tabulation and supervised the project. All authors reviewed and approved the final revision of the manuscript.

## Appendix: description of AIS codes to the Marshall Classes cross-tabulation

### AIS codes outside cerebrum or unrelated to raised ICP

The Marshall Classification enables categorizing a subset of TBI patients at risk of developing intracranial hypertension. Therefore injuries sustained in the brain stem and cerebellum are ignored. However, there are many codes describing the injuries in these two anatomical locations in the AIS dictionary. Almost all these codes do not have a Marshall Class equivalent. Bearing this in mind, we differentiated other non cerebral injuries relating to the brain stem or cerebellum by grouping them as 'brain stem injury' or 'cerebellar injury' without allocating a Marshall Class. The exception is AIS code 140202, which identifies the brain stem compression and thus corresponds to the Marshall Class III which involves compressed or absent cisterns. Moreover, the Marshall System is not designed for penetrating injuries [3] for which there are several AIS codes. Therefore, we grouped all such AIS codes as "penetrating injury" with no Marshall Class allocated. However, a penetrating injury AIS code related to the brain stem or cerebellum should still be grouped as penetrating injury rather than a cerebellar or brain stem injury. This is because penetrating and blunt brain injuries pathophysilogically differ.

Similarly, a number of AIS codes representing cerebral injuries are not directly related to raised intracranial pressure. These include massive destruction of both cranium and brain (crush), infarction, intraventricular hemorrhage, ischaemia, pneumocephalus, laceration and pituitary injury. Such injuries are best mapped to the Marshall Class II since they do not indicate a normal CT (i.e. Marshall Class I) nor do they indicate brain swelling or mass lesions (i.e. Marshall Classes III and above). However, crush injury should be mapped to the most severe Marshall Class i.e. Class VI because of the very severe nature of this injury.

### Not Further Specified (NFS) AIS codes

In allocating the appropriate Marshall Class to the cerebral AIS codes, we assumed that NFS injuries are minimally severe injuries of their type as is always the case in the dictionary. For example, the code 140999 which represents cerebral NFS was allocated to Marshall Class II, which represents the least severe brain injury in the Marshall Classification.

### Brain swelling

Although, in the Marshall Classification, only class III is declared as 'brain swelling' by Marshall et. al., class IV also contains this pathology. This is because midline shift, which denotes class IV, can be caused by brain swelling as well. Thus, there are two Marshall Classes of III and IV indicating brain swelling, which are distinguished by compressed/absent cisterns for class III and midline shift of more than 5 mm for class IV. However, in AIS coding the degree of the brain swelling is determined by the status of ventricles/cisterns being normal, compressed or absent. Therefore, the highest degree of brain swelling in AIS dictionary, i.e. absent cisterns, actually falls in the Marshall Class that indicates the lowest degree of brain swelling (class III) with no equivalent AIS code for Marshall Class IV. This inability in the AIS dictionary to distinguish between Marshall Classes III and IV poses a problem. The decision is whether or not to pool all AIS codes of brain swelling into Marshall Class III and to leave Class IV blank or to allocate AIS codes of mild and moderate brain swelling to Marshall Class III and AIS codes for severe swelling to Class IV. We selected the second option assuming that Marshall Classes III and IV represent mild and severe brain swelling respectively, irrespective of the criteria of the severity.

### Mass lesions

There are several separate AIS codes for two kinds of mass lesions; contusion and hemorrhage. There are also several severity groups (small, moderate, large, massive or extensive) into which these lesions can fall depending on the size as ascertained by AIS severity scores. Furthermore, the cut-offs for this classification based on size are different in the AIS dictionary and the Marshall Classification. Whilst the Marshall Classification uses the simple cut-off of 25 cc regardless of type and location, those used in the AIS dictionary vary by the type, anatomical location and, at times, by age of the patient. For instance, a single contusion in the cerebrum is small when < 30 cc, large when between 30 cc and 50 cc and is extensive when > 50 cc (the cut-offs for the size-wise grouping of intracerebral hemorrhage, epidural or subdural hematoma are receptively 30 cc, 50 cc and 50 cc).

Regarding the size of high density mass lesions, a problem exists on the cut-off or criteria to distinguish small from large lesions being different in the AIS dictionary and the Marshall Classification. Therefore the assumption was made that small haemorrhage and contusion (unilateral or bilateral), SAH and Subpial haemorrhage correspond to the Marshall Class II with all other large, massive or extensive mass lesions coming under class VI.

### Skull fractures

Codes indicating several skeletal fractures (*basal skull fracture *or *not simple vault fractures) *were all placed in Marshall Class I, which is described as no intracranial pathology.

### AIS 2005

Adapting our proposed mapping for the 2005 update is simple since we know that the update to the head section involves changes in a number of AIS scores and the addition of some new codes. None of the old AIS codes, which have undergone changes in their severity score, are affected in terms of their mapped Marshall Class. Regarding the new codes, some have arisen because some of the old AIS codes have been further sub-divided to specify the injuries in more detail. Overall these criteria do not affect the mapping proposed in Table 2 for each particular injury. For example, in the 2005 AIS dictionary, the severity of Diffuse Axonal Injury (DAI) is further qualified by whether or not it is confined to white matter/basal ganglia or involves the corpus callusom. No matter which is the case, the equivalent Marshall Class II, as allocated in Table 2, still holds. Nevertheless, there are 3 new codes (140701, 140702 and 140703) that describe the hypoxic or ischemic brain damage which occurs due to systemic hypoxia, hypotension or shock. Since these causes of brain damage are not directly related to head trauma, we can infer that the head CT of such patients should be clear which indicates Marshall Class I (no visible pathology).

## Pre-publication history

The pre-publication history for this paper can be accessed here:

http://www.biomedcentral.com/1471-2288/10/72/prepub
